# Cellular stress responses in protein misfolding diseases

**DOI:** 10.4155/fso.15.42

**Published:** 2015-09-01

**Authors:** Martin L Duennwald

**Affiliations:** 1 Department of Pathology & Laboratory Medicine, Schulich School of Medicine & Dentistry, University of Western Ontario, London, Ontario, Canada

**Keywords:** cellular protein quality control, heat shock response, polyQ expansion diseases, protein misfolding, stress response pathways, unfolded protein response

## Abstract

Many human diseases, particularly neurodegenerative diseases, are associated with protein misfolding. Cellular protein quality control includes all processes that ensure proper protein folding and thus prevent the toxic consequences of protein misfolding. The heat shock response (HSR) and the unfolded protein response (UPR) are major stress response pathways within protein quality control that antagonize protein misfolding in the cytosol and the endoplasmic reticulum, respectively. Huntington's disease is an inherited neurodegenerative disease caused by the misfolding of an abnormally expanded polyglutamine (polyQ) region in the protein huntingtin (Htt), polyQHtt. Using Huntington's disease as a paradigm, I review here the central role of both the HSR and the UPR in defining the toxicity associated with polyQHtt in Huntington's disease. These findings may begin to unravel a previously unappreciated cooperation between different stress response pathways in cells expressing misfolded proteins and consequently in neurodegenerative diseases.

**Figure 1.**
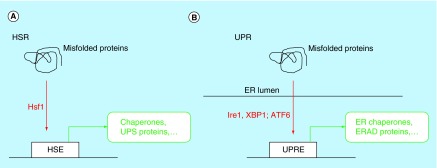
**Key signaling and transcriptional events.** Events in **(A)** the HSR and **(B)** the UPR. ER: Endoplasmic reticulum; ERAD: Endoplasmic reticulum-associated protein degradation; HSE: Heat shock element; HSR:Heat shock response; UPR:Unfolded protein response; UPRE:Unfolded protein response elements; UPS: Ubiquitin proteasome system.

**Figure 2.**
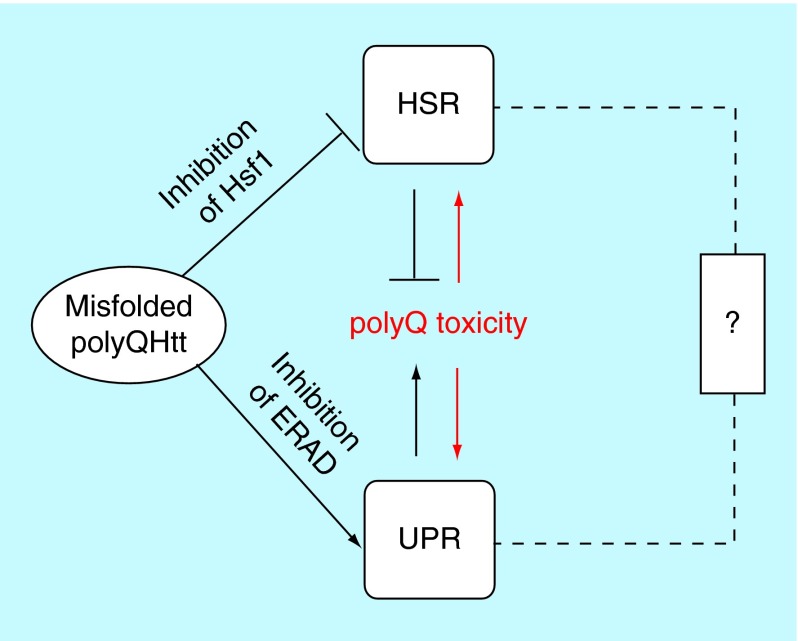
**The heat shock response and the unfolded protein response act together in exacerbating polyQ toxicity in Huntington's disease.** The ‘?’ indicates a possible albeit hitherto mostly unexplored direct connection between the HSR and the UPR. ERAD: Endoplasmic reticulum-associated protein degradation; HSR: Heat shock response; UPR: Unfolded protein response.

All proteins have to attain their accurate 3D conformation in order to function properly, a process termed protein folding. When protein folding goes awry, a process termed protein misfolding, the affected proteins either lose their regular function, gain new toxic functions or both. Accordingly, protein misfolding is associated with many human diseases, particularly neurodegenerative diseases, such as Alzheimer's disease, Parkinson's disease and Huntington's disease [1].

Cellular protein quality control encompasses all cellular processes that direct the proper production, maintenance and turnover of proteins [2]. Multiple stress response pathways regulate cellular protein quality control to prevent the detrimental effects of proteins misfolding. The heat shock response (HSR) is elicited by protein misfolding in the cytosol, whereas the unfolded protein response (UPR) is elicited by protein misfolding in the endoplasmic reticulum (ER). Apparently, these and possibly other stress response pathways, fail in neurodegenerative diseases, thus unmasking or exacerbating the cytotoxicity caused by protein misfolding. Recent studies have begun to unravel an intricate connection between protein misfolding and both the HSR and the UPR in neurodegenerative diseases, particularly in Huntington's disease.

## Huntington's disease & other polyglutamine expansion diseases

Huntington's disease (HD) is caused by an expansion of the CAG triplet repeat (encoding for the amino acid glutamine, Q) in the first exon of the IT15 gene, which encodes the protein huntingtin (Htt) [3]. The regular (i.e., not disease associated) function of huntingtin remains mostly unclear but it has been suggested to perform a role as a scaffolding protein coordinating the interactions between many proteins in different cellular pathways [4]. While a loss of this regular function of Htt may contribute to HD, it is predominantly the gain of toxic function of polyglutamine (polyQ) expanded Htt that elicits neurodegeneration [5]. Accordingly, many experimental models of HD have been created by simply expressing full length or a small amino-terminal fragments of polyQ expanded Htt. These experimental models include yeast, worms, flies, mice, rats, monkeys and cultured mammalian cells, including neurons, all of which recapitulate major hallmarks of polyQ proteins, including their polyQ length-dependent aggregation and toxicity.

Eight neurodegenerative diseases in addition to HD are caused by abnormal polyQ expansions, all of which are inherited in a dominant manner [6]. In all of these diseases, the length of the polyQ expansion directly correlates with the severity of the disease progression and inversely correlates with the onset of disease. Yet the associated polyQ expansions are found in distinct proteins and each of polyQ expansion diseases presents a distinct neuropathology, affecting different regions and neurons in the brain, which results in distinct symptoms. Thus, polyQ expansion diseases share general disease mechanisms, foremost protein misfolding, while other features specify each disease. The cellular bases of these disease-specific features remain mostly unclear. HD affects primarily medium spiny neurons in the striatum but also neurons in the cortex and to a lesser extent other brain regions [7,8]. The mechanisms underlying this cell type specific toxicity of polyQ expanded Htt are also poorly understood.

Studies in HD models have established a daunting number of cellular pathways that are disturbed by polyQ expanded Htt, most of which have been validated in brain samples from HD patients. These pathways include transcription regulation [9], axonal transport [10], endocytosis [11], mitochondrial metabolism [12] and protein degradation [13]. It remains a major challenge to establish when each of these defects occur during HD pathogenesis and hence discern primary cellular defects that initiate the disease process from secondary effects that merely reflect a degenerating cellular state. It is, however, evident that the misfolding of polyQ expanded Htt presents one of the primary and earliest pathological events in HD.

## PolyQHtt misfolding & aggregation in Huntington's disease

Highly stable microscopically detectable polyQHtt inclusions, or aggregates are pathological hallmarks of HD [14]. Yet polyQHtt misfolding is not restricted to polyQHtt aggregation. In fact, the views of the role of polyQHtt aggregates in HD are evolving. Aggregates are found both in the cytosol and in the nucleus of neurons in brains from HD patients and nuclear aggregates have been suggested to be the major toxic species [15]. Many studies provide convincing evidence that polyQHtt aggregates can be toxic and can impair essential cellular processes. Efforts to find treatments for HD have therefore focused on reducing polyQ aggregation as a main therapeutic target [16]. By contrast, other studies provide equally convincing evidence that polyQHtt aggregation correlates with reduced polyQ toxicity [17]. Indeed, smaller and less stable assemblies of polyQHtt, for example, polyQHtt oligomers, can be highly toxic, whereas bigger aggregates may be benign or even perform protective functions. Consequently, reducing polyQ aggregation in HD could increase polyQHtt oligomer concentrations, thus increasing toxicity. Augmenting the formation of protective aggregates has consequently been explored as an alternative therapeutic approach [18]. Clearly, further research will have to clarify the perplexing role of polyQ aggregation in HD and thus assess its potential as a therapeutic target.

Cellular protein quality control, primarily molecular chaperones and protein remodeling factors modulate the assembly and disassembly of protein aggregates including polyQHtt aggregates, but also modulate other misfolded proteins species including polyQHtt oligomers. Also, many proteins of the cellular protein quality control machinery possess the capacity to protect cells from the toxic effects of polyQHtt misfolding, whether aggregated or oligomeric. This raises the question whether this protection does not occur at all, is insufficient, or fails in HD. In other words, it remains mostly unclear how cellular protein quality control directs polyQHtt misfolding, aggregation and the resulting toxicity during the course of HD pathogenesis.

## The heat shock response

The expression of many molecular chaperones and protein remodeling factors is controlled by the HSR. The HSR is a conserved cellular response to various stresses, including exposure to elevated temperatures, heavy metals and, generally, the presence of misfolded proteins in the cytosol (Figure 1 A). The HSR is primarily regulated by the Heat shock transcription factor 1, Hsf1 [19]. Upon activation, for example, exposure to high temperatures, Hsf1 translocates from the cytosol to the nucleus and binds as a homotrimer to heat shock elements (HSEs), which are promoter consensus sequences that regulate the expression of heat shock genes. Hsf1 activation also involves Hsf1 phosphorylation, dephosphorylation and deacetylation, and changing interactions with molecular chaperones, such as Hsp90, Hsp40 and Hsp70 [20]. A dissociation of molecular chaperones (mostly Hsp90) from Hsf1 has been speculated to be an initiating event during Hsf1 activation. The HSR induces the expression of many proteins involved in cellular stress response, including the aforementioned molecular chaperones and protein remodeling factors and proteins of the ubiquitin proteasome system (UPS). In addition, the HSR induces the expression of genes involved in energy metabolism and other cellular pathways that do not appear to be directly linked to cellular protein quality control [21].

## The heat shock response in Huntington's disease

We and others have explored the link between the HSR, Hsf1 and polyQHtt toxicity. Starting with studies in yeast, we found that the HSR is not induced by the presence of misfolded polyQHtt [22]. In fact, polyQHtt expressing yeast cells have an impaired HSR when exposed to elevated temperatures. These results were confirmed in mammalian cell lines that either express full-length polyQ expanded Htt or an amino-terminal fragment of polyQ expanded Htt [23]. In both of these HD models, cells expressing polyQ expanded Htt are highly sensitized to a heat shock and other HSR inducing conditions. This increased sensitivity is at least partially caused by the inability of cells expressing polyQHtt to elicit a proper HSR. Small molecules that induce the HSR, such as inhibitors of the molecular chaperone Hsp90, have the capacity to ameliorate polyQ Htt toxicity implying that the HSR and Hsf1 modulate polyQHtt aggregation and toxicity in HD [24]. Labbadia *et al*. showed that an Hsp90 inhibitor can reduce polyQ toxicity even though the HSR is strongly impaired in HD mouse models compared with wild-type mice [25]. Furthermore, Kopito *et al*. demonstrated that the activation and overexpression of Hsf1 enhances polyQHtt aggregation [26]. Even though this study did not directly assess polyQHtt toxicity, its findings suggest a protective role of polyQHtt aggregation, which is regulated by Hsf1 and the HSR.

Experiments in HD mouse and cell models further assessed the defect in Hsf1 activation in cells expressing polyQHtt [23,25]. This Hsf1 activation defect did not seem to correlate with any obvious changes in any of the multiple Hsf1 activation steps, since all of these (e.g., translocation to the nucleus, trimerization and post-translational modifications) appeared intact in heat-shocked polyQHtt expressing cells. We did, however, observe reduced overall protein levels of Hsf1 in polyQHtt expressing cells, even under regular (nonstressed) growth conditions [23]. We confirmed these reduced Hsf1 levels in mouse brains of HD knock-in mice.

How does polyQHtt alter Hsf1-dependent gene expression? We found that Hsf1's binding to target promoter regions and the Hsf1-dependent expression profiles changed profoundly when exposed to heat shock in cells expressing full-length polyQHtt [27]. These polyQHtt-associated changes not only included anticipated HSR genes, such as molecular chaperones and protein remodeling factors, but also the expression of genes that do not seem directly linked to cellular protein quality control, such as genes encoding cytoskeletal proteins or focal adhesions proteins. The Labbadia *et al*. study suggests alterations in chromatin architecture as a possible explanation of the impaired HSR in HD [25].

Taken together, these results demonstrate that the HSR through Hsf1 modulate polyQHtt aggregation and toxicity. These studies also indicate that polyQHtt alters Hsf1 activation and the HSR. Thus, it is plausible to speculate that this HSR/polyQHtt interaction creates a vicious feed-forward cycle, in which the presence of polyQHtt first impairs the HSR, thus exacerbating polyQ toxicity by producing more toxic protein species (e.g., oligomers), which in turn impair the HSR even further. Disrupting this vicious cycle, for example, by eliciting the HSR with small molecules – either early in the disease process or even before disease onset – might therefore be a promising therapeutic target for the treatment of HD.

## The unfolded protein response

The UPR is the cellular stress response to protein misfolding in the ER [28]. The failed assembly of protein complexes, impaired protein degradation, impaired formation of disulfide bonds, and impaired protein glycosylation in the ER are common triggers of the UPR (Figure 1 B). Protein misfolding in the ER is sensed by the membrane protein Ire1. Ire1 homo-oligomerizes and then phosphorylates other Ire1 proteins. Activated Ire1 then facilitates the alternative splicing of the mRNA of the transcription factor Xbp1. The alternative spliced Xbp1 message is translated into active Xbp1 proteins, which triggers the expression of genes that bear UPR response elements in their promoter regions. In addition to Ire1, ER stress activates the transport of the protein ATF6 from the ER to the Golgi, where it is cleaved, translocates to the nucleus, and induces the expression of UPR genes. The UPR controls the expression of ER molecular chaperones, proteins involved in the degradation of ER proteins (ERAD) and proteins involved in lipid synthesis. ER stress also induces the phosphorylation of the kinase PERK, which attenuates protein biosynthesis of secretory proteins, thus temporarily lowering the burden of protein folding in the ER.

## The unfolded protein response in Huntington's disease

ER stress and the induction of the UPR are well-established in HD models and HD patient samples [29,30]. The sequestration of essential proteins of the ERAD complex by polyQHtt causes the accumulation of misfolded proteins in the ER and thus UPR induction [22]. Also, cells expressing polyQHtt are highly sensitive to ER stress, such as disturbed protein glycosylation or altered ER calcium levels. Furthermore, our studies in yeast and HD cell models showed that constitutive activation of the UPR reduces polyQ toxicity [22]. These results suggest that continuous ER stress and the resulting continuous and unattenuated induction of the UPR contribute to HD pathogenesis.

## Two stress responses are affected by polyQHtt & modulate its toxicity in Huntington's disease

As outlined above, two major cellular stress response pathways, the HSR and the UPR, are affected by polyQHtt. While polyQHtt weakens the HSR, it elicits an overactive and cytotoxic UPR. How do these two disturbances of cellular protein quality control join forces in causing polyQ toxicity? Possibly, the impaired HSR produces elevated species of misfolded polyQHtt that cause higher levels of ER stress, thus eliciting the UPR. Supporting this notion, the Lederkremer group showed that essential ERAD proteins are indeed more efficiently sequestered by soluble polyQHtt species rather than by insoluble ones [31]. Consequently, the vicious cycle of perpetual impairment of the HSR progressively increases ER stress, which might eventually lead to neuronal dysfunction and eventually neuronal death (Figure 2).

The HSR and the UPR are ubiquitous stress responses that function in every cell in the human body, including every neuron. Why then in HD does polyQHtt so explicitly affect distinct neurons in a specific region of the brain, for example, the striatum? Several studies indicate that specific neurons in the striatum are highly sensitive to ER stress. Future research comparing different classes of neurons in HD-affected and nonaffected regions of the brain will have to clarify why these neurons are particularly vulnerable to disturbances of both the HSR and the UPR. Also, comparing HD with other polyQ expansion diseases and the role of the HSR and the UPR in the neurons that are most affected in each disease might help to elucidate the idiosyncratic nature of polyQHtt toxicity in HD. We propose that is the specific disturbance of both the HSR and the UPR and that primarily affects striatal neurons in HD.

## Coordinated stress responses?

The HSR and the UPR are usually regarded as independently regulated stress response pathways that are triggered by distinct cellular stressors. As documented above, however, the misfolding of polyQHtt can make these two pathways functionally interact in what could be interpreted as a coordinated yet failing attempt to modulate polyQ misfolding and toxicity. It is thus conceivable that other protein misfolding events or stress conditions may prompt a similar coordinated interplay between the HSR and the UPR. Notably, some genes bear both HSEs and UPR response elements in their prompter regions and are thus transcriptionally regulated by both the HSR and the UPR [32,33]. Also, exposing cells to prolonged yet lower nonlethal levels of stress, which may be highly relevant to many physiological and pathological scenarios, have begun to unravel a link between the HSR and the UPR. Hence, it may be productive to view the HSR and the UPR, and possibly other cellular stress response pathway, as functioning in a coordinated manner within cellular protein quality control, particularly in the context of protein misfolding diseases. Future therapeutic strategies might have to take into account this interconnected nature of cellular protein quality control to effectively reduce the toxicity associated with protein misfolding and thus delay disease onset or slow down disease progression.

## Conclusion

In conclusion, both the HSR and the UPR play a crucial and possibly coordinated role in HD pathogenesis. Future research will have to unravel the molecular mechanisms connecting these two cellular stress response programs in order to utilize them as therapeutic targets for the treatment of HD

**Executive summary**

Polyglutamine huntingtin (polyQHtt) misfolds and forms aggregates in Huntington's disease (HD).
PolyQHtt aggregates in HD may perform toxic, benign or even protective functions.
Activating the heat shock response (HSR) can reduce polyQHtt toxicity and modulates its misfolding and aggregation.
Cells expressing polyQHtt are sensitized to heat shock and have an impaired HSR.
PolyQHtt inhibits ER proteins (ERAD) and thus elicits the unfolded protein response.
HSR and unfolded protein response are linked in regulating polyQHtt toxicity.
